# Glucagon-like peptide-1 and dual/triple receptor agonists in the treatment of metabolic dysfunction-associated steatotic liver disease: advances in mechanistic research

**DOI:** 10.3389/fmed.2026.1763185

**Published:** 2026-02-12

**Authors:** Xinyi Lu, Li Yang

**Affiliations:** 1 The Second School of Clinical Medicine, Zhujiang Hospital, Southern Medical University, Guangzhou, Guangdong, China; 2 Department of Endocrinology, Zhujiang Hospital, Southern Medical University, Guangzhou, Guangdong, China

**Keywords:** fibrosis, glucagon-like peptide-1 receptor agonists, inflammation, metabolic dysfunction-associated steatohepatitis, metabolic dysfunction-associated steatotic liver disease

## Abstract

Metabolic Dysfunction-Associated Steatotic Liver Disease (MASLD) has emerged as a prevalent and severe global hepatic disorder, necessitating the development of effective therapeutic strategies. Glucagon-like peptide-1 receptor agonists (GLP-1RAs), along with glucose-dependent insulinotropic polypeptide (GIP) and glucagon (GCG) dual or triple receptor agonists that modulate multiple metabolic pathways, have attracted significant scientific interest due to their multifaceted roles in metabolic regulation. This review provides a comprehensive overview of the mechanistic insights into the effects of GLP-1RAs and dual or triple receptor agonists on the pathophysiology of MASLD, with a focus on hepatic lipid metabolism, inflammatory responses, and fibrosis progression.

## 1 Introduction

Metabolic dysfunction-associated steatotic liver disease (MASLD), formerly referred to as non-alcoholic fatty liver disease (NAFLD), has become the most prevalent chronic liver condition worldwide, affecting approximately one-third to nearly 40% of the adult population globally, with projections indicating an increase to over 55% by 2040 (1, 2). The clinical spectrum of MASLD spans from simple steatosis, which is often benign and potentially reversible, to metabolic dysfunction-associated steatohepatitis (MASH), a progressive and inflammatory condition that may lead to hepatic fibrosis, cirrhosis, and hepatocellular carcinoma (HCC) (2, 3). There is a critical unmet need for effective pharmacotherapies that target the underlying metabolic and inflammatory mechanisms driving MASLD. Resmetirom, a selective liver thyroid hormone receptor *β* (THR-β) agonist, received approval from the FDA in 2024 for the treatment of adult patients with F2-F3 MASH (4).

Glucagon-like peptide-1 receptor agonists (GLP-1RAs) have gained considerable attention in recent years as promising therapeutic agents for MASLD, particularly owing to their well-established efficacy in treating type 2 diabetes mellitus (T2DM) and obesity, conditions closely implicated to MASLD pathogenesis (2, 5). Clinical trials, including phase 2 and 3 studies, have reported significant improvements in liver histology, including MASH resolution and decreased hepatic fat content, with GLP-1RA therapy (6, 7). Subsequently, in August 2025, semaglutide, a representative drug of GLP-1RAs, also obtained FDA approval, targeting the same indications as Resmetirom (8). Building on the therapeutic success of GLP-1RAs, recent pharmacological developments have introduced GLP-1 with glucose-dependent insulinotropic polypeptide (GIP)/glucagon (GCG) dual or triple receptor agonists simultaneously target multiple incretin pathways, offering a novel multi-targeted approach to MASLD treatment.

**Figure 1:**
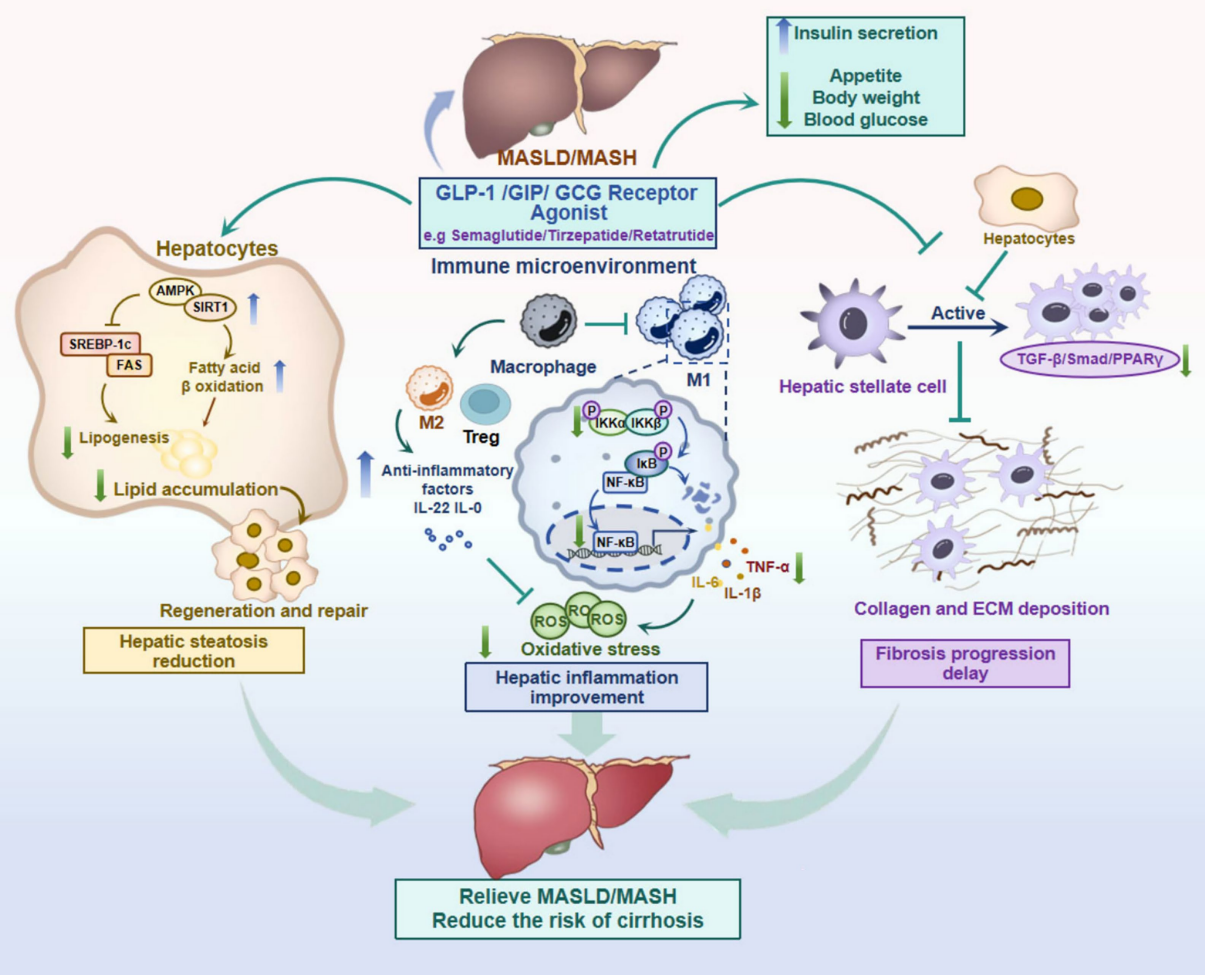
The mechanistic insights of GLP-1 with GIP/GCG dual or triple receptor agonists in MASLD treatment. Created with BioRender.com. Top right illustration: GLP-1RAs regulate hepatic lipid metabolism via indirect systemic effects. Left panel: Lipid metabolism regulation within hepatocytes: GLP-1 RA, by activating the AMPK/SIRT1 pathway, promote fatty acid oxidation (*β*-oxidation) and inhibit lipogenesis (via suppression of SREBP-1c/FAS), thereby fundamentally reducing lipid accumulation in hepatocytes. Middle panel: Anti-inflammation and immune regulation within immunity microenvironment: GLP-1 RAs inhibits the NF-κB signaling pathway, a key mediator of inflammation, thereby reducing the production of pro-inflammatory cytokines such as TNF-*α* and IL-6 by macrophages in macrophage (M1) and other immune cells. Concurrently, it enhances the expression of anti-inflammatory factors, including IL-22/IL-10 in Treg and macrophage (M2), contributing to the mitigation of hepatic inflammation and oxidative stress. Right panel: Anti-fibrotic and protective effects within HSCs: GLP-1RAs directly inhibit hepatic stellate cell activation, reducing the TGF-β/Smad/PPARγ signaling pathway, resulting in decreased collagen deposition and delayed progression of fibrosis. MASH, Metabolic Dysfunction-Associated Steatohepatitis; MASLD, Metabolic Dysfunction-Associated Steatotic Liver Disease; GLP-1 RAs, Glucagon Like Peptide-1 Receptor Agonists; GIP, Glucose-dependent Insulinotropic Polypeptide; GCG, Glucagon; AMPK/SIRT1, AMP-activated Protein Kinase/Sirtuin-1; SREBP-1c/FAS, Sterol Regulatory Element-Binding Protein 1c/Fatty Acid Synthase; NF-κB, Nuclear Factor kappa-B; TNF-α, Tumor Necrosis Factor alpha; IL-1β/6/10/22, Interleukin-1β6/10/22; Treg, Regulatory T cell; Macrophage1/2, M1/M2; TGF-β/Smad, Transforming Growth Factor-beta; PPARγ, Peroxisome Proliferator-Activated Receptor gamma; ECM, Extracellular Matrix.

This review aims to systematically summarize the mechanistic insights and clinical advances of GLP-1with dual or triple receptor agonists in MASLD treatment, thereby promoting a more profound comprehension and facilitating their application in clinical practice (Figure 1).

## 2 MASLD’s pathophysiology and current treatment

MASLD is characterized by an abnormal accumulation of lipids in hepatocytes, commonly occurring in the context of obesity, insulin resistance, and T2DM-key features of metabolic syndrome that substantially affect patient quality of life and long-term prognosis (1). The pathophysiology of MASLD is complex and multifactorial, involving hepatic lipid accumulation driven by excess adiposity, systemic inflammation, insulin resistance, and activation of hepatic stellate cells (HSC)-factors that collectively contribute to liver injury and fibrogenesis. Traditionally, diagnosis has depended on invasive liver biopsy; meanwhile, noninvasive imaging modalities and serum biomarkers are now increasingly helpful for both diagnosis and disease monitoring (1).

The complex pathophysiology of MASLD calls for integrated, multi-faceted therapeutic regimens and improved diagnostic tools to enhance clinical outcomes. Several agents have demonstrated histological and biochemical effects by targeting core pathophysiological pathways. Recently, Resmetirom, a thyroid receptor agonist was approved by the FDA as the first drug on the label for MASH with fibrosis 2 and 3. At the moment, some medications can collaborate in the treatment of MASLD, some of them approved for the treatment of comorbidities such as GLP-1 agonists indicated for the treatment of T2DM or obesity, but also with benefit in the MASH treatment (9, 10). Also Phase three studies are underway for MASLD with these drugs. Pioglitazone and vitamin E can be considered with improvement in MASH and stabilization of fibrosis, as suggested by some studies (10, 11).

## 3 The mechanisms of GLP-1 with dual or triple receptor agonists in MASLD treatment

### 3.1 The mechanisms of GLP-1 receptor agonists in MASLD treatment

#### 3.1.1 Regulation of hepatic lipid metabolism

GLP-1RAs exert multifaceted regulatory effects on hepatic lipid metabolism, which are pivotal to their therapeutic potential in MASLD. A common thread among new treatments is the activation of energy- sensing pathways, especially AMP- activated protein kinase (AMPK), which helps the liver break down fatty acids, stops lipogenesis and reduces inflammation and fibrosis. Experimental evidence clearly demonstrates that GLP-1RAs such as liraglutide and semaglutide enhance AMPK activity, leading to increased expression of genes involved in mitochondrial fatty acid oxidation and diminished lipogenesis (12, 13). This metabolic alteration not only decreases intrahepatic triglyceride content but also alleviates lipotoxicity, a key driver of hepatocellular injury in MASLD.

Concomitantly, GLP-1RAs suppress the expression of lipogenic transcription factors and enzymes, including sterol regulatory element-binding protein 1c (SREBP-1c) and fatty acid synthase (FAS), resulting in reduced *de novo* lipogenesis. For instance, *in vitro* studies using HepG2 cells treated with palmitic acid revealed that the GLP-1RA exendin-4 downregulated the expression of SREBP-1c and FAS, thereby attenuating steatosis (14, 15). This regulatory effect is partly mediated via the AMPK/sirtuin-1 (SIRT1) axis, as knockdown of SIRT1 abrogated the lipid-lowering and autophagy-inducing effects of GLP-1RAs, highlighting a crucial role of this pathway in regulating lipid metabolism by facilitating degradation of lipid droplets, thus preventing excessive lipid accumulation (12). Moreover, GLP-1RAs enhance the export of lipids from hepatocytes, contributing to restoration of lipid homeostasis (16). Clinical studies provide strong support for these mechanistic insights, showing that GLP-1RA treatment significantly reduces liver fat content (LFC) as measured by imaging modalities and improves biochemical markers of liver injury (17, 18). Meta-analyses of randomized controlled trials (RCTs) reveal that GLP-1RAs decrease hepatic steatosis by approximately 4–5% and improve histological features of steatohepatitis (7, 19). The hepatic benefits of GLP-1RAs are partially independent of weight loss, as demonstrated in clinical trials in which matched weight loss achieved by lifestyle intervention did not replicate the full metabolic improvements seen with GLP-1RA therapy, including enhanced glucose handling and decreased *de novo* lipogenesis (20).

In summary, GLP-1 receptor agonists regulate hepatic lipid metabolism in MASLD through activation of AMPK-mediated fatty acid *β*-oxidation, suppressing lipogenesis-related gene expression, and enhancing lipid export and autophagy. This indicates that GLP-1RAs are effective agents for the management of MASLD.

#### 3.1.2 Anti-inflammatory and immunomodulatory effects

Chronic hepatic inflammation, driven by activated Kupffer cells, infiltrating macrophages, and pro-inflammatory cytokines like tumor necrosis factor-alpha (TNF-*α*) and interleukin-6 (IL-6), is a crucial factor in MASLD progression to steatohepatitis and fibrosis. Beyond metabolic regulation, GLP-1RAs exhibit potent anti-inflammatory and immunomodulatory properties that contribute to their therapeutic efficacy in MASLD. Preclinical studies have clearly demonstrated that GLP-1RAs effectively reduce hepatic macrophage infiltration and suppress expression of TNF-*α*, IL-6, and other pro-inflammatory mediators in animal models of MASLD (21, 22). This effect is, at least in part, mediated through the inhibition of the nuclear factor-kappa B (NF-κB) signaling pathway, which serves as a central regulator of inflammatory gene transcription. Treatment with GLP-1RA downregulates NF-κB activation in hepatic tissue, thereby leading to decreased transcription of cytokines and chemokines that sustain inflammation (23, 24).

Furthermore, GLP-1RAs influence the phenotype and function of immune cells. They promote a shift from pro-inflammatory M1 macrophages to anti-inflammatory M2 macrophages, thereby creating a reparative hepatic environment (25). Additionally, GLP-1RAs augment regulatory T cell (Treg) populations and elevate the levels of anti-inflammatory cytokines such as IL-10, contributing to immune tolerance and resolution of inflammation (26). These immunomodulatory effects are not confined to the liver. Clinical studies have demonstrated that GLP-1RAs can reduce systemic inflammation markers, including C-reactive protein (CRP) and IL-18Rα (27, 28). Mechanistic insights also reveal that GLP-1RAs modulate inflammasome activity, specifically the NLRP3 inflammasome, thus reducing pyroptosis and inflammatory cell death in hepatic and immune cells (29).

In conclusion, GLP-1 receptor agonists effectively alleviate hepatic inflammation in MASLD by suppressing pro-inflammatory cytokine release, inhibiting NF-κB signaling, modulating the phenotypes of immune cells, and reducing the activation of inflammasomes. These immunomodulatory effects complement their metabolic actions, contributing to improved liver outcomes and systemic benefits.

#### 3.1.3 Anti-fibrotic and hepatoprotective effects

Fibrosis serves as a crucial of prognosis in MASLD, with progressive extracellular matrix deposition leading to cirrhosis and liver failure. Activated HSCs are responsible for the production of collagen and other matrix components. Mechanistically, GLP-1RAs inhibit the activation of hepatic stellate cells (HSCs), which are the principal fibrogenic cell type in the liver. Transforming growth factor-*β* (TGF-β) plays a pivotal role in the fbrotic process and is considered a key driver of fibrosis. Treatment with GLP-1RAs reduces expression of *α*-smooth muscle actin (α-SMA) and collagen type I in HSCs, thereby attenuating fibrogenesis (18, 30). This effect is mediated through modulation of signaling pathways TGF-*β*/Smad and peroxisome proliferator-activated receptor gamma (PPARγ), which regulate HSC activation and extracellular matrix (ECM) synthesis (30, 31).

*In vivo* studies conducted on animal models of MASLD/MASH have reveal that GLP-1RAs can reduce liver fibrosis scores and collagen deposition, accompanied by decreased inflammatory infiltration and oxidative stress (24, 32). Clinical trials have reported improvements in noninvasive fibrosis markers, including liver stiffness measured by elastography and serum biomarkers such as procollagen III peptide, following GLP-1RA therapy (18, 33). Although histological reversal of advanced fibrosis remains challenging, GLP-1RAs seem to prevent the progression of fibrosis and may promote the regression of early-stage fibrosis.

Beyond anti-fibrotic effects, GLP-1RAs promote hepatocyte regeneration and repair. They enhance autophagy and reduce lipotoxicity-induced hepatocyte apoptosis, thereby preserving the integrity of liver parenchyma (12). GLP-1RAs also improve hepatic microcirculation and reduce oxidative stress, contributing to hepatoprotection (29). Combination therapies that involve GLP-1RAs and other agents, such as fibroblast growth factor 21 (FGF21) analogs, THR-*β* action to enhance anti-fibrotic efficacy (5, 34).

In summary, GLP-1 receptor agonists exert anti-fibrotic effects in MASLD through the inhibition of hepatic stellate cell activation, the modulation of profibrotic signaling pathways, and the promotion of hepatocyte regeneration. This clearly highlights GLP-1RAs as promising agents for the management of MASLD.

### 3.2 The mechanisms of GLP-1/GIP or GCG receptor dual agonists in MASLD

#### 3.2.1 Metabolic regulatory effects

Dual agonists targeting GLP-1 and GIP receptors have emerged as promising agents for metabolic regulation in MASLD. The GIP receptor agonism complements GLP-1 receptor activation by enhancing glucose-dependent insulin secretion, thereby improving glycemic control and weight loss more effectively than GLP-1RAs alone. Tirzepatide’s imbalanced receptor engagement, which favors GIP receptor activation, combined with biased signaling at the GLP-1 receptor, further enhances insulin secretion and metabolic benefits (35, 36). These multifaceted metabolic effects position dual GLP-1/GIP receptor agonists as superior therapeutic candidates for MASLD, especially in patients with coexisting obesity and T2DM, where dysregulated glucose and lipid metabolism drive the progression of the disease.

#### 3.2.2 Regulation of hepatic lipid metabolism

Dual GLP-1 receptor agonists facilitate hepatic lipid metabolism through coordinated activation of intracellular signaling pathways that suppress lipogenesis and enhance fatty acid oxidation. The GLP-1 component activates hepatic AMPK/SIRT1 pathways, which inhibit SREBP-1c, a master regulator of *de novo* lipogenesis, thereby reducing hepatic triglyceride synthesis (37, 38). Concurrently, the GCGR agonist component stimulates the hepatic cyclic AMP (cAMP)/protein kinase A (PKA) pathway, robustly enhancing mitochondrial *β*-oxidation of fatty acids and ketogenesis. This activation mobilizes stored triglycerides within hepatocytes for oxidation, effectively decreasing hepatic lipid content (39, 40). The dual action synergistically suppresses hepatic *de novo* lipogenesis while promoting lipid catabolism, resulting in a net reduction of hepatic steatosis (41, 42). Animal studies have demonstrated that combination therapies involving GLP-1 receptor agonists and agents targeting lipid metabolism pathways produce marked reductions in liver fat content, downregulate lipogenic enzymes, and upregulate proteins involved in lipid oxidation (41).

In summary, this dual mechanism addresses the fundamental lipid dysregulation in MASLD by restoring the balance between lipid synthesis and degradation within the liver, thereby mitigating steatosis and its metabolic consequences (40, 43).

#### 3.2.3 Anti-inflammatory and anti-fibrotic effects

Activation of GLP-1 receptors by dual agonists exerts potent anti-inflammatory effects within the liver microenvironment, which are crucial for preventing progression from steatosis to steatohepatitis and fibrosis. GLP-1 receptor activation inhibits the activation of hepatic Kupffer cells and infiltrating macrophages, leading to decreased production of pro-inflammatory cytokines such as TNF-*α* and IL-1β, thereby attenuating hepatic inflammation (44).

Preclinical models have shown that GLP-1 analogs suppress hepatic stellate cell activation, the key drivers of fibrogenesis, resulting in reduced collagen deposition and fibrosis (45, 46). Additionally, GCGR activation may indirectly influence fibrogenesis by modulating the hepatic metabolic milieu, reducing lipotoxicity and oxidative stress that serve as upstream triggers for fibrotic pathways (39, 47). By alleviating lipotoxicity and inflammatory signaling, dual GLP-1 receptor agonists disrupt the feed-forward cycle driving hepatic fibrosis, thus offering a therapeutic avenue to prevent or reverse liver fibrosis in MASLD (40, 48).

Clinical trials that investigate dual GLP-1/GIP receptor agonists have presented encouraging evidence for their efficacy and safety in treating MASLD. Phase 2 and 3 studies, such as randomized controlled trials, have shown that these agents significantly reduce liver fat content, improve liver enzyme profiles, and promote histological improvements in patients with MASLD and MASH (6, 46). In a randomized, placebo-controlled study, pemvidutide, a dual GLP-1/GCG receptor agonist, significantly decreased liver fat content by up to 68.5% after 12 weeks of treatment, accompanied by reductions in hepatic inflammation markers and body weight, highlighting the potential of dual receptor agonists to modify MASLD pathophysiology (47). Meta-analyses have further confirmed that dual GLP-1/GIP receptor agonists are more effective than single GLP-1RAs in reversing liver fibrosis and reducing hepatic fat accumulation, suggesting a dose-dependent and receptor-combinatorial effect on liver outcomes (48).

This integrated anti-inflammatory and anti-fibrotic action complements their metabolic benefits, positioning dual GLP-1 receptor agonists as promising agents in the comprehensive management of MASLD and its complications.

### 3.3 The mechanistic GLP-1/GIP/GCG receptors triple agonists in MASLD

#### 3.3.1 Metabolic effects

The development of triple receptor agonists targeting GLP-1, GIP, and GCG receptors represents a promising advancement in the treatment of MASLD. Its triple-agonist mechanism achieves multi-pathway metabolic regulation by simultaneously activating GLP-1 receptors (enhancing insulin secretion and inhibiting gastric emptying), GIP receptors (promoting energy storage in adipose tissue), and GCG receptors (increasing energy expenditure). At present, seven triple receptor agonists, including Retatrutide (LY3437943), SAR441255 and xGLP/GCG/GIP-32, have entered the clinical research stage. Among them, retatrutide has shown significant clinical advantages in phase II clinical trials, reducing the weight of obese patients by up to 24.2% in 48 weeks and lowering the HbA1c of type 2 diabetes patients by 2.2% in 36 weeks (49, 50). The combination of these three hormones results in a weight loss effect that surpasses that achieved by individual or dual agonists, making it a cornerstone in the therapeutic approach to MASLD (6).

#### 3.3.2 Regulation of hepatic lipid metabolism

Triple receptor agonists exhibit promising synergistic effects on hepatic lipid metabolism, a central pathological feature in MASLD. These hormones exert direct effects on the liver; glucagon receptor activation stimulates *β*-oxidation of fatty acids, reducing hepatic lipid content, while GLP-1 enhances systemic insulin sensitivity and decreases lipolysis in adipose tissue, thereby indirectly reducing hepatic fat influx (51). In a phase 2 trial in participants with obesity and MASLD, once-weekly treatment with the GIP/GLP-1/GCG triple agonist retatrutide resulted in substantial reductions in liver fat, body weight, abdominal visceral adipose tissue (VAT) and abdominal subcutaneous adipose tissue (ASAT), serum lipids. Hepatic steatosis resolved in more than 85% of participants in two highest dose groups (52). These mechanisms suggest a coordinated regulation of key enzymes and signaling pathways involved in lipid synthesis and breakdown, such as AMPK and SREBP-1c, which are critical for maintaining hepatic lipid homeostasis (6).

#### 3.3.3 Anti-inflammatory and anti-fibrotic effects

The anti-inflammatory and anti-fibrotic potential of GLP-1 receptor activation has garnered significant attention in the context of liver disease. Activation of GLP-1 receptors has been shown to exert direct anti-inflammatory effects, potentially by inhibiting the activation of hepatic stellate cells and reducing the production of pro-inflammatory cytokines (6). This action may be particularly beneficial in the context of MASLD, where inflammation plays a pivotal role in disease progression. The combination of GLP-1, GIP, and glucagon receptor agonism may further enhance these effects by collectively reducing the hepatic fat burden and systemic inflammatory state, thereby indirectly inhibiting the progression of fibrosis (47). In preclinical models with an acute diet-induced liver injury model due to a single high-dose fructose binge in mice, as well as a chronic mouse feeding model incorporating fructose intended to mirror early MASLD with features of steatohepatitis, Retatrutide reduced the expression of hepatic inflammation markers chemokine ligand-1 (Cxcl-1), serum amyloid A1 (Saa-1), and serum amyloid A2 (Saa-2) (53). Retatrutide markedly suppressed the expression of pro-inflammatory cytokines (TNF-*α*, caspase-1, and NLRP3) and pro-fibrotic factors (fibronectin, α-SMA, and collagen I) in treating diabetic kidney disease (DKD) in db/db mice (54). Furthermore, Retatrutide substantially enhanced liver function, reduced triglyceride levels, cholesterol levels. The potential for these triple receptor agonists to not only improve metabolic parameters but also to exert protective effects on liver inflammation and fibrosis positions them as promising candidates for future therapeutic strategies in managing metabolic liver diseases.

In summary, triple receptor agonists orchestrate a multidimensional regulation of liver inflammation and fibrosis by modulating cytokine profiles, inhibiting fibrogenic signaling, and improving metabolic derangements that contribute to hepatic injury. This comprehensive and targeted approach presents a novel therapeutic avenue that effectively addresses both the metabolic and histopathological facets of MASLD.

## 4 Challenges and future directions

### 4.1 Safety considerations

The safety profile of dual or triple receptor agonists targeting GLP-1, GIP, and GCG receptors in the treatment of MASLD and related metabolic disorders warrants careful evaluation. The glucagon component, while contributing to enhanced metabolic benefits, poses a potential risk of hypoglycemia, necessitating precise dose titration to avoid adverse glycemic events. Clinical trials have reported gastrointestinal side effects as common adverse events with incretin-based therapies, including nausea and vomiting, which may affect patient adherence. Meanwhile, it is worth noting that although the current data do not show a significant increase in the risk of pancreatitis, long-term follow-up should still focus on the potential risks of pancreatitis and thyroid C-cell tumors, especially as GCGR activation may affect pancreatic exocrine function (55). Moreover, individual metabolic characteristics, such as coexisting diabetes, obesity, or hepatic impairment, influence drug metabolism and response, underscoring the need for personalized treatment strategies.

### 4.2 Combination therapy strategies

Combining triple receptor agonists with other pharmacological agents holds promise for synergistic metabolic benefits in MASLD management. Notably, co-administration with farnesoid X receptor (FXR) agonists or peroxisome proliferator-activated receptor (PPAR) agonists may enhance lipid metabolism regulation and anti-inflammatory effects, addressing multiple pathogenic pathways simultaneously. Preclinical and clinical data suggest that FXR and PPAR dual modulators can improve insulin sensitivity, reduce hepatic steatosis, and attenuate fibrosis, which may complement the actions of GLP-1/GIP/GCG receptors agonists. Additionally, sodium-glucose cotransporter 2 (SGLT2) inhibitors, known for their glycemic and cardiovascular benefits, when combined with incretin-based therapies, have demonstrated additive improvements in weight loss, glycemic control, and liver function markers. Optimizing dosing schedules and administration routes is critical to maximize therapeutic efficacy while minimizing adverse effects such as hypoglycemia and gastrointestinal intolerance. Nonetheless, rigorous clinical trials are needed to establish the safety, efficacy, and optimal protocols for these combination regimens in MASLD patients (56–58).

## 5 Conclusion

In conclusion, the evolving landscape of MASLD treatment underscores the significant therapeutic promise of GLP-1RAs and dual or triple receptor agonists. From an expert perspective, the development of GLP-1RAs has marked a pivotal advancement by targeting key pathophysiological mechanisms such as hepatic lipid metabolism, inflammation, and fibrosis. These agents have demonstrated robust efficacy in modulating metabolic dysfunctions central to MASLD progression, positioning them as a cornerstone in contemporary therapeutic strategies. Continued interdisciplinary research that integrates basic science, clinical investigation, and personalized medicine principles will be crucial for fully harnessing their therapeutic potential and improving outcomes for patients suffering from MASLD.
